# Vitamin D Deficiency in COVID-19 Patients and Role of Calcifediol Supplementation

**DOI:** 10.3390/nu15153392

**Published:** 2023-07-30

**Authors:** Christian Mingiano, Tommaso Picchioni, Guido Cavati, Filippo Pirrotta, Marco Calabrese, Ranuccio Nuti, Stefano Gonnelli, Alberto Fortini, Bruno Frediani, Luigi Gennari, Daniela Merlotti

**Affiliations:** 1 Department of Medicine Surgery and Neuroscience, University of Siena, 53100 Siena, Italy; mingiano2@student.unisi.it (C.M.); guido.cavati@student.unisi.it (G.C.); pirrotta@student.unisi.it (F.P.); m.calabrese5@student.unisi.it (M.C.); ranuccio.nuti@unisi.it (R.N.); stefano.gonnelli@unisi.it (S.G.); bruno.frediani@unisi.it (B.F.); luigi.gennari@unisi.it (L.G.); 2 Internal Medicine Unit, Ospedale San Giovanni di Dio, 50143 Florence, Italy; tommaso.picchioni@uslcentro.toscana.it (T.P.); alberto.fortini@uslcentro.toscana.it (A.F.); 3 Department of Medical Sciences, Azienda Ospedaliera Universitaria Senese, 53100 Siena, Italy

**Keywords:** vitamin D deficiency, 25-OH vitamin D, COVID-19, calcifediol, vitamin D supplementation, SARS-CoV-2

## Abstract

Hypovitaminosis D has been associated with worse outcome in respiratory tract infections, with conflicting opinions regarding its role in Coronavirus-19 disease (COVID-19). Our study aimed to evaluate the possible relationship between 25-OH vitamin D (25OHD) values and the following conditions in patients hospitalized for COVID-19: prognosis, mortality, invasive (IV) and non-invasive (NIV) mechanical ventilation, and orotracheal intubation (OTI). A further objective was the analysis of a possible positive effect of supplementation with calcifediol on COVID-19 severity and prognosis. We analyzed 288 patients hospitalized at the San Giovanni di Dio Hospital in Florence and the Santa Maria alle Scotte Hospital in Siena, from November 2020 to February 2021. The 25OHD levels correlated positively with the partial pressure of oxygen and FiO2 (PaO2/FiO2) ratio (r = 0.17; *p* < 0.05). Furthermore, when we analyzed the patients according to the type of respiratory support, we found that 25OHD levels were markedly reduced in patients who underwent non-invasive ventilation and orotracheal intubation (OTI). The evaluation of the length of hospitalization in our population evidenced a longer duration of hospitalization in patients with severe 25OHD deficiency (<10 ng/mL). Moreover, we found a statistically significant difference in the mortality rate between patients who had 25OHD levels below 10 ng/mL and those with levels above this threshold in the total population (50.8% vs. 25.5%, *p* = 0.005), as well as between patients with 25OHD levels below 20 ng/mL and those with levels above that threshold (38.4% vs. 24.6%, *p* = 0.04). Moreover, COVID-19 patients supplemented with calcifediol presented a significantly reduced length of hospitalization (*p* < 0.05). Interestingly, when we analyzed the possible effects of calcifediol on mortality rate in patients with COVID-19, we found that the percentage of deaths was significantly higher in patients who did not receive any supplementation than in those who were treated with calcifediol (*p* < 0.05) In conclusion, we have demonstrated with our study the best prognosis of COVID-19 patients with adequate vitamin D levels and patients treated with calcifediol supplementation.

## 1. Background

Coronavirus-19 disease (COVID-19) is a contagious coronavirus-related syndrome that has been causing a pandemic since December 2019. The responsible pathogen was named severe acute respiratory syndrome coronavirus 2 (SARS-CoV-2) by the International Committee on Taxonomy of Viruses (ICTV) [1]. COVID-19 has a variable clinical picture, ranging from severe to asymptomatic cases. Certainly, an important role is played by comorbidities that aggravate the clinical picture and worsen the prognosis. The most relevant prognostic factors are older age, cardiovascular diseases, diabetes, hypertension, chronic lung diseases, onco-hematological pathology, renal failure, obesity, and smoking [2]. Other negative prognostic factors are a high Sequential Organ Failure Assessment (SOFA) score; a D-dimer of >1 µg/mL; high levels of IL-6, serum ferritin, troponin I, and lactate dehydrogenase (LDH); and reduced lymphocyte serum levels [3]. Moreover, a greater risk of death has been observed in males than in females [4,5]. Despite the advancement of knowledge regarding the pathogenesis of the virus, most available therapies have been proven to be insufficient and to have variable efficacy, particularly before the development of vaccines. Considering this, our interest has turned to the analysis of the role that vitamin D deficiency could have in patients hospitalized for COVID-19 and the possible impact that oral intake of calcifediol could have on prognosis and mortality in those subjects. Vitamin D is a fat-soluble vitamin essential in bone and calcium–phosphorus homeostasis and has several other functions, many of which are still not completely understood [6]. The vitamin D receptor (VDR) is expressed in tissues and cells of almost the whole body, such as the intestinal tract, kidneys, parathyroid glands, bones, immune cells, keratinocytes, and bronchial and gastrointestinal epithelial cells [7]. Through interaction with its VDR, calcitriol regulates the transcription of numerous genes [8]. Numerous clinical studies have shown an association between vitamin D deficiency and an increased risk of infections, especially of the upper respiratory tract, and the beneficial effect in preventing those infections with supplementation of vitamin D [9,10]. In this regard, vitamin D plays an important immunomodulant role. In particular, macrophages, monocytes, and dendritic cells have both the VDR and CYP27B1 enzyme, meaning that those cells are able to locally convert calcifediol (25-hydroxy vitamin D or 25OHD) into its active form, calcitriol (1,25OH2D) [11], and therefore they can regulate their own response, maturation, differentiation, and chemotactic activity [12,13,14]; moreover, via this pathway, those cells negatively regulate the production of proinflammatory cytokines, such as IL6, while inducing the formation of anti-inflammatory cytokines, such as IL-10 [15,16]. Recently, some studies have indicated that vitamin D seems to be able to stimulate the production of some antimicrobial molecules such as defensins and cathelicidins, which seems to play an important role in Sars-CoV-2 infection, simultaneously blocking the viral Spike 1 protein (S1) and cloaking angiotensin-converting enzyme-2 (ACE2) [17,18]. In addition, calcitriol acts directly on T-lymphocytes, inhibiting the proliferation and differentiation of Th1 and Th17 cells, favoring Th2, therefore increasing the production of anti-inflammatory cytokines such as IL-5 and IL-10 [19,20,21]. A similar action has been shown in B-lymphocytes; calcitriol interferes directly with their differentiation into plasma cells, decreasing the production of antibodies and increasing the production of IL-10 [22]. Numerous studies have focused on the hypothesis of a correlation between a more severe form of COVID-19 and hypovitaminosis D, with conflicting results [23,24,25,26,27,28,29,30]. In a previous meta-analysis including 1403715 COVID-19 patients from 54 studies performed until March 31th 2021, we showed that patients with hypovitaminosis D presented an increased risk of severe acute respiratory syndrome due to SARS-CoV-2 infection, requiring admission to an intensive care unit and increasing in-hospital mortality [29]. Similar results were found by other systematic reviews and meta-analyses [31,32,33]. Numerous studies examined the possible positive effects of vitamin D supplementation in patients with SARS-CoV-2 infection. A meta-analysis of three different studies (two randomized controlled studies and one retrospective case–control study) included 189 patients who were supplemented with vitamin D and 343 patients who were treated with standard care. A lower ICU hospitalization rate was observed in patients with vitamin D supplementation than in patients receiving standard care (OR: 0.36; 95% CI: 0.210–0.626). Regarding mortality, vitamin D supplements produced comparable results to a placebo or usual treatment (OR: 0.93; 95% CI: 0.413–2.113; *p* = 0.87) [34]. A different systematic review of three randomized clinical trials including 356 participants, of whom 183 were treated with vitamin D, demonstrated benefits in terms of mortality and mechanical ventilation; however, both results need to be confirmed by RCTs that can overcome the heterogeneity in terms of population and the type and dose of vitamin D supplementation [35]. Moreover, a large observational study of 838 patients hospitalized for COVID-19, of whom 447 were treated with calcifediol (532 μg on day 1 + 266 μg on days 3, 7, 15, and 30) and compared with the other 391 patients who were not supplemented, showed an increased risk of transfer to the ICU and mortality in patients not treated with calcifediol, considering age, sex, and comorbidities [36]. Taking into account these observations, our study aimed to provide further insight into the possible role of vitamin D status in prognosis and mortality in a large and well-characterized cohort of patients hospitalized for SARS-CoV-2 infection, specifically investigating the need for non-invasive ventilation (NIV), orotracheal intubation (OTI), and the clinical outcome. Moreover, taking into account a threshold 25OHD level of 20 ng/mL for vitamin D deficiency, we also investigated the effects of 25OHD (calcifediol) on COVID-19 severity and prognosis.

## 2. Materials and Methods

### 2.1. Study Population

Our study population included 288 patients, from the ‘San Giovanni di Dio Hospital’ in Florence and the ‘Santa Maria alle Scotte University Hospital’ in Siena, hospitalized for COVID-19 from November 2020 to May 2021. This time frame was specifically selected in order to avoid the possible interference of SARS-CoV-2 vaccination in the clinical outcomes of COVID-19. Upon access to the COVID-19 department, a detailed medical history was collected, and a complete physical examination was carried out on all patients. Most of patients referred the beginning of symptoms related to SARS-CoV-2 infection requiring medical care within 7 days prior hospitalization. Vital parameters and arterial blood gas (ABG) were measured. All patients underwent laboratory investigations, consisting of blood count with formula; liver and kidney function; coagulation profile (prothrombin time (PT) and activated partial thromboplastin time (aPTT); fibrinogen and D-dimer; inflammation parameters (C-reactive protein, CRP), ferritin and interleukin-6 (IL-6), lactate dehydrogenase (LDH), N-terminal pro-brain natriuretic peptide (NT-proBNP), troponin, 25-hydroxy vitamin D (25OHD), procalcitonin (PCT)); and finally serology (IgG and IgM) for SARS-CoV-2. At hospital admittance, electrocardiogram (ECG), chest ultrasound, and compression ultrasonography (CUS) of the lower limbs were performed. The Charlson comorbidity index was also calculated for each patient. Accordingly, given the clinical manifestations and disease evolution, and at the discretion of the examining physician, further diagnostic investigations were performed in a subset of cases, such as chest computed tomography (CT) and echocardiography. Informed consent was obtained from all subjects involved in the study.

### 2.2. 25OHD Measurement

Vitamin D status was assessed by the measurement of total 25OHD levels with ELISA methodology (DiaSorin Diagnostics, Saluggia, Italy; sensitivity, 1.5 ng/mL; interassay CV: <11%, intraassay CV: <12.5%). The quality and accuracy of the 25OHD analysis from our laboratory was validated on an ongoing basis by participation in the External Quality Evaluation Program from the “Centro di Riferimento per la Qualità dei Servizi di Medicina di Laboratorio” (http://www.qualitalaboratorilombardia.it/public/razionale_ormoniemarcatoritumorali.pdf. Accessed on 19 June 2023). According to the Endocrine Society thresholds defining vitamin D status [37], the following thresholds for vitamin D status were considered: (1) 25OHD values below 10 ng/mL (severe vitamin D deficiency); (2) 25OHD values below 20 ng/mL (vitamin D deficiency); (3) 25OHD values below 30 ng/mL (vitamin D insufficiency); and (4) 25OHD values above 30 ng/mL.

In order to assess the effects of SARS-CoV-2 infection on circulating 25OHD levels in a limited subset of cases, the measurement of 25OHD levels was repeated after 5 and 10 days from admission without receiving any supplementation with calcifediol. Moreover, information on 25OHD levels assessed in the same year before admission (mean: 5.9 ± 3.3 months) was also collected when available.

### 2.3. Calcifediol Supplementation

Since 7 December 2020 (following the local ethical committee approval), patients with 25OHD levels below 20 ng/mL at hospital admittance were treated within 72 h of admittance with 2 boluses of calcifediol of 450 micrograms each for 2 consecutive days for a total of 900 micrograms. This dosage was selected in order to achieve a rapid normalization of 25OHD levels and was based on the positive results from a previous study with high calcifediol doses [36,38].

### 2.4. Statistical Analysis

Data are expressed as means (± standard deviation) or median (interquartile range) for continuous variables and as absolute values (percentages) for discrete variables. Chi-square test was used to test for differences between groups for non-parametric variables. To compare the biochemical parameters, such as the serum concentration of 25OHD between the various groups of subjects, analysis of variance (ANOVA) and covariance (ANCOVA) were used, stratifying by various cofactors (e.g., sex and age). The correlation between the variables was evaluated through the analysis of partial and simple correlation coefficients according to ‘’Pearson Statistica 5.1′’ software for Windows (Statsoft Inc., Tulsa, OK, USA) and SPSS software for Windows (Statistical Package for Social Science, SPSS Ltd., Chicago, IL, USA), which were used to perform the statistical analyses in the sample. We considered a *p*-value < 0.05 to be significant.

## 3. Results

**Figure 1:**
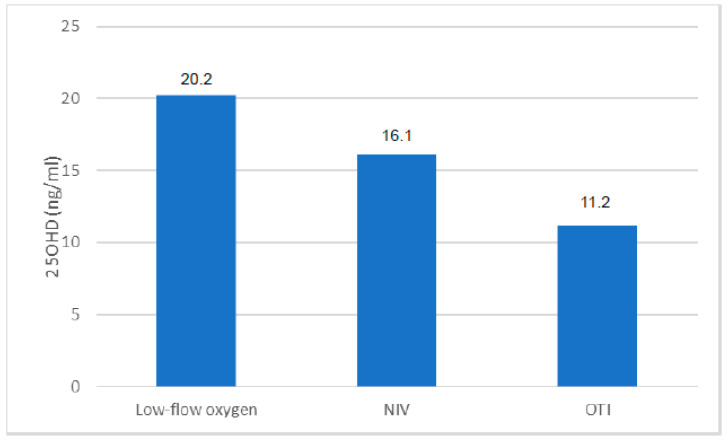
Serum levels of 25OHD in COVID-19 patients according to ventilation support. NIV = non-invasive ventilation support; OTI = orotracheal intubation.

**Figure 2:**
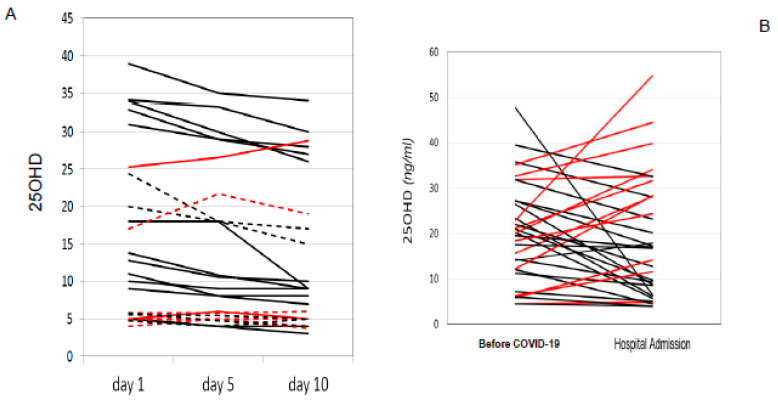
25OHD serum levels in COVID-19 patients during hospitalization (**A**) and information on 25OHD levels assessed in the same year before admission (**B**). Red lines indicate an increase in 25OHD levels while black lines indicate no change or a decrease in 25OHD levels. Dotted lines indicate deceased patients.

**Figure 3:**
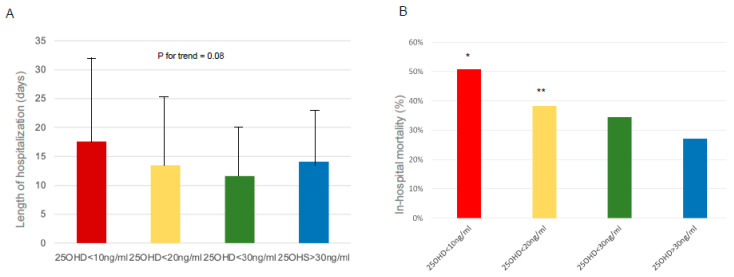
Length of hospitalization (**A**) and in-hospital mortality rate (**B**) in COVID-19 patients according to serum levels of 25OHD; * *p* = 0.005 considering patients with 25OHD levels < 10 ng/mL compared with those with 25OHD levels > 10 ng/mL; ** *p* = 0.04 considering patients with 25OHD levels <20 ng/mL compared with those with 25OHD levels >20 ng/mL.

In the selected time frame, 288 patients were included in the study, of whom 56 received calcifediol supplementation due to 25OHD levels below 20 ng/mL. During their hospital stay, 82 patients died from COVID-19 complications. Men represented 66% of the total population (n = 190), of which 24% died during hospitalization (n = 46), while women represented only 34% of the sample (n = 98) with a prevalence of death of 37% (n = 36). The prevalence of 25OHD insufficiency (<30 ng/mL) in the total population analyzed was 83% (n = 239), with no statistically significant differences between the two genders. 25OHD levels at admittance in the COVID-19 area were inversely but not significantly associated with age (r = −0.09, *p*-value = 0.1313) and BMI (r= −0.14, *p*-value = 0.15). Moreover, we did not find any significant differences in 25OHD levels in relation to the following comorbidities: arterial hypertension, diabetes mellitus, cardiovascular disease, Chronic Obstructive Pulmonary Disease (COPD), and grade IIIb-V Chronic Renal Failure (CRF) (data not shown). However, 25OHD levels tended to be lower in the presence of multiple comorbidities. We then investigated the correlations between the circulating levels of 25OHD and the serum biochemical or arterial blood gas parameters evaluated at hospital admittance in the total population. We found a significant negative correlation between 25OHD and fibrinogen (r = −0.16; *p*-value = 0.05) or lactate dehydrogenase (LDH) levels (r = −0.16; *p*-value = 0.05). Moreover, the levels of 25OHD were positively correlated both with the Spo2/FIO2 (r= 0.28; *p* < 0.05) and PaO2/FiO2 ratio (r = 0.17; *p* < 0.05). Interestingly, when we analyzed patients according to the type of respiratory support, we did not find significant differences in 25OHD levels, albeit a slight reduction was evident in patients who underwent non-invasive ventilation (High-Flow Nasal Cannula (HFNC), Continuous Positive Airway Pressure (CPAP), and bilevel ventilation system) and orotracheal intubation (OTI) (Figure 1). In the subset of cases in which the measurement of 25OHD levels was repeated after 5 and 10 days from admission, we found a slight decrease in 25OHD levels, particularly during the first 5 days of hospitalization (Figure 2A). Similarly, the information on 25OHD levels assessed in the same year before admission in the same patients revealed that there was also the same trend of decreasing levels before infection (Figure 2B), thus suggesting that COVID-19 infection did not consistently impact vitamin D status in our cohort of patients. After excluding deceased patients, a longer duration of hospitalization was observed in patients with severe 25OHD deficiency (<10 ng/mL) than in those with 25ODH deficiency (<20 ng/mL), insufficiency (<30 ng/mL), or normal values (>30 ng/mL) (Figure 3A). We then focused our attention on the effect of COVID-19 infection and mortality in our population. The general characteristics of the deceased or alive patients are shown in Table 1. We found that deceased patients were older than survivors and had more comorbidities, as documented by a significantly increased Charlson comorbidity index. Table 2 shows the laboratory findings measured at hospital admission in both categories. As is evident, deceased patients showed a significant reduction in hemoglobin levels, plasma cells, and lymphocyte count and an increased neutrophil count compared with survivors, while, as expected, renal function, prothrombotic state, and inflammation markers were significantly altered in deceased subjects. Moreover, a slight but not significant decrease in 25OHD levels was observed in deceased patients compared with the group of survivors. This trend was also confirmed when we separately considered patients who received vitamin D supplementation at home before hospital admittance versus those who did not receive any supplementation. Interestingly, when we considered the different thresholds of 25OHD deficiency, we found a statistically significant difference in the mortality rate between patients who had 25OHD levels below 10 ng/mL (indicative of severe vitamin D deficiency) and those with levels above this threshold in the total population (*p* = 0.005), as well as between patients with 25OHD levels below 20 ng/mL (indicative of vitamin D deficiency) and those with levels above that threshold (*p* = 0.04). Indeed, we generally found that the in-hospital mortality rate was inversely correlated to the degree of 25OHD deficiency (Figure 3B).

### Effects of Calcifediol Supplementation

**Figure 4:**
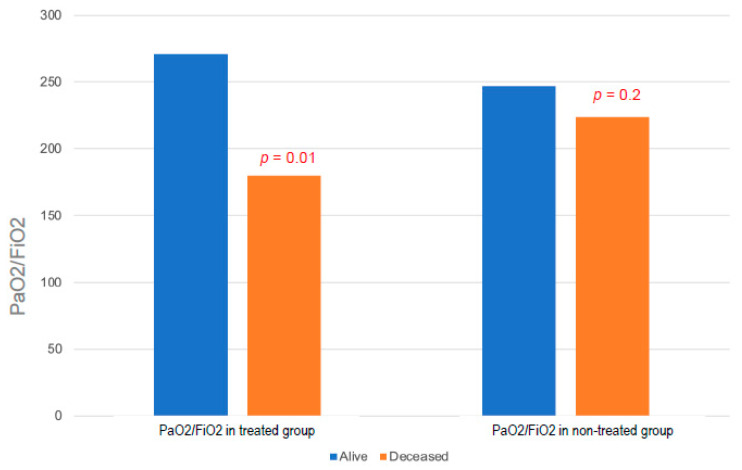
PaO2/FiO2 ratio differences in alive and deceased COVID-19 subjects according to calcifediol supplementation. PaO2/FiO2: ratio of arterial oxygen partial pressure/fraction of inspired oxygen.

**Figure 5:**
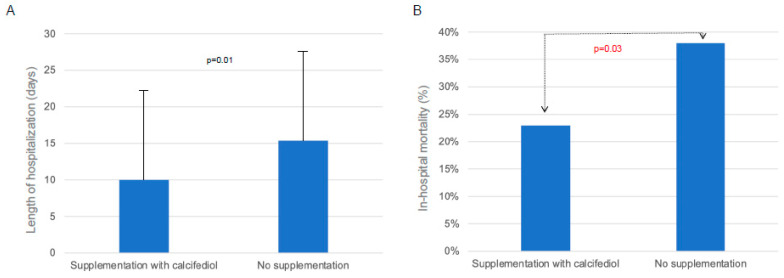
Length of hospitalization (**A**) and in-hospital mortality (**B**) in COVID-19 patients supplemented with calcifediol.

The general characteristics of COVID-19 patients according to calcifediol supplementation are shown in Table 3. We did not find any significant difference in biochemical or respiratory parameters between the two groups. However, the PaO2/FiO2 ratio (as the expression of impaired respiratory function) did not differ among deceased or alive patients who did not receive calcifediol supplementation, while a severe PaO2/FiO2 reduction was observed in calcifediol-supplemented patients who were deceased compared with supplemented patients who survived (Figure 4). Moreover, when we analyzed the effect of calcifediol treatment on respiratory support we found that only 32% of supplemented patients required NIV during hospitalization vs. 42% of patients not treated with calcifediol, although this difference was not statistically significant (*p* = 0.2). Then, we considered the possible effect of calcifediol supplementation on the length of hospitalization, and we found a significantly shorter duration of hospital stay in supplemented subjects (*p* = 0.01) (Figure 5A). Interestingly, when we analyzed the possible effects of calcifediol on mortality rate in patients with COVID-19, we found that the percentage of deaths was significantly higher in patients who did not receive any supplementation than in those who were treated with calcifediol (*p* = 0.03) (Figure 5B).

## 4. Discussion

The dramatic evolution of the SARS-CoV-2 pandemic, together with its high morbidity and mortality rates, prompted us to investigate the possible mechanisms that lead to serious clinical outcomes in an attempt to identify effective treatments. In this context, we measured 25OHD levels in a well-characterized cohort of patients admitted to hospital for SARS-CoV-2 infection from the first and second pandemic waves. Our study confirmed the presence of a high prevalence of 25OHD insufficiency in patients hospitalized for SARS-CoV-2; in fact, 87% of patients had levels below 30 ng/mL. Interestingly, other broader studies, including cohort studies, have reported the presence of significantly lower vitamin D levels in COVID-19 patients than in healthy subjects, thus suggesting a relationship between insufficient vitamin D levels and SARS-CoV-2 positivity [39]. Importantly, because we measured total 25OHD levels at hospital admittance for SARS-CoV-2 infection, we cannot completely rule out the possibility that our findings may be due to reverse causation, i.e., that acute illness may have led to a reduction in the total levels of 25OHD through suppression of the vitamin D binding protein or other mechanisms. In fact, because the 25OHD levels were measured when patients were hospitalized, lower 25OHD levels can be expected in those patients because vitamin D status can be affected by acute inflammation, particularly decreasing in cases of severe infection [40]. Indeed, it is still controversial whether or not free 25OHD levels are a better predictor than total 25OHD levels for health outcomes [41,42]. However, other studies have evaluated the relationship between 25OHD levels measured prior to SARS-CoV-2 infection and vitamin D deficiency and COVID-19 severity and have shown a similar association [43]. This is consistent with what we observed in the subset of patients who underwent multiple measurements of 25OHD levels, namely a slight decrease in 25OHD levels, particularly during the first 5 days of hospitalization. Likewise, we did not observe a significant decrease in 25OHD levels in the same year before admission due to SARS-CoV-2 infection, suggesting that COVID-19 infection did not consistently impact vitamin D status in our cohort of patients. In the overall sample, circulating levels of 25OHD were inversely correlated with increasing serum levels of inflammation markers (CRP, D-dimer, fibrinogen), reaching statistical significance for LDH. Interestingly, low vitamin D status has previously been shown to be associated with an enhanced acute inflammatory reaction which becomes particularly relevant when 25OHD levels are below 20 ng/mL [38,39,40,44,45,46]. In our study, low levels of 25OHD were associated with low values of the SpO2/FiO2 ratio evaluated at admittance in the COVID-19 area. This relationship is closely related to the degree of severity of respiratory distress, thus confirming a possible role of 25OHD in protecting the respiratory system from viral infection, as demonstrated by some studies in the literature [9]. We did not demonstrate a statistically significant difference in 25OHD levels between the deceased group of individuals and the survivors. However, by subdividing the patients according to the severity of vitamin D deficiency, statistical significance was achieved regarding the increase in the mortality rate in patients in whom the deficiency was more severe. The highest difference was observed in patients with severe deficiency (<10 ng/mL), in whom the mortality rate was about double that of patients with higher levels, similar to data reported in a series of studies in Northern Italy [47]. However, we have highlighted statistically significant differences in lower 25OHD levels between subjects with greater respiratory impairment and individuals without severe respiratory distress, further confirming a possible correlation between vitamin D status and clinical severity of COVID-19. Since 7 December 2020, patients with vitamin D deficiency (25OHD levels below 20 ng/mL calcifediol), including COVID-19 patients, were supplemented with calcifediol, considering the pharmacokinetic and functional characteristics of this compound and its superiority in rapidly optimizing 25OHD levels [48]. We found a positive effect of calcifediol supplementation on the length of hospitalization, similar to what already described that found in [49], confirming that the clinical outcome of COVID-19 patients requiring hospitalization could be improved by administration of vitamin D, which is expected to obtain a sustained serum 25OHD concentration even above the 30 ng/mL threshold, necessary to attain a robust immune system for overcoming infections [44,45,50,51]. In addition, when we compared deceased subjects with survivors in the subgroup of calcifediol-treated patients, the levels of PaO2/FiO2 were lower in the deceased subgroup, indicating a more compromised respiratory and clinical status at admittance. Conversely, this difference was not present in the control group. Regarding the hypothesis of a therapeutic effect of boluses with calcifediol, these data could suggest that the deceased patients, despite the treatment, presented a more advanced clinical status of the disease, in which the use of calcifediol was insufficient, or more likely administered too late, and therefore unuseful for improving the prognosis. These results have numerous limitations deriving from the small number of patients in our study, particularly concerning the group of patients supplemented with calcifediol. Another limitation of this study is the lack of multiple 25OHD dosages in all the enrolled patients, including patients supplemented with calcifediol. This could provide further insights on the optimal 25OHD levels to be achieved. Moreover, the lack of monitoring of the lymphocyte percentage and the decrease in the neutrophil-to-lymphocyte ratio prevented a better assessment of the possible immunomodulatory effect of vitamin D supplementation [52]. However, our study population represents a large and well-characterized cohort of COVID-19 patients before vaccination, in which the role of vitamin D could be more relevant. Nevertheless, other studies are needed to confirm the prognostic value of 25OHD levels in patients suffering from COVID-19 and the role of any benefit deriving from supplementary therapy with calcifediol.

## 5. Conclusions

Despite the described limitations, in our cohort of hospitalized COVID-19 patients we found a remarkably high prevalence of vitamin D deficiency compared with the general population. Furthermore, low levels of 25OHD were associated with increased levels of serum markers of inflammation, which in turn seemed to have a negative predictive role regarding the course of this infectious disease. The high mortality rate found in patients with severe deficiency confirmed the possibility that low serum levels of vitamin D may be associated with a negative prognosis and clinical outcome; in fact, we showed a higher prevalence of deficiency of 25OHD in individuals who underwent invasive respiratory support than in those who underwent NIV. The data on the lower in-hospital mortality of patients treated with calcifediol are strengthened by the homogeneity of the two groups (treated vs. controls) in terms of age, comorbidities, and BMI. Finally, the patients who died in the treated group were already more compromised, which was demonstrated by the parameters of admission to the hospital; therefore, such a critical clinical status may have reduced the effectiveness of calcifediol supplementation, which may have been administered too late. Therefore, these data deserve attention since, even without considering the possible causal link between vitamin D deficiency and the clinical course of COVID-19, the finding of low levels of 25OHD in patients with severe COVID-19 could at least be considered as a prognostic marker, thus also confirming the immunoprotective role of vitamin D and its preventive role in COVID-19 infection when administered as a supplement [50]. In conclusion, taking into account the low cost and the almost total lack of safety problems related to supplementation with vitamin D, well-designed interventional studies are needed to explore whether vitamin D replacement is effective in reducing clinical severity and preventing the risk of respiratory failure in patients with infection due to SARS-CoV-2, and eventually other pathogens as suggested in the other studies available to date [33,35,36,48,51].

## Figures and Tables

**Table 1:** General characteristics of study populations. Values are expressed as mean value (±SD). M: males. F: females. BMI: Body Mass Index.

Characteristics	Alive (n = 206)	Deceased (n = 82)	*p*-Value
Age (yrs)	69.4 (±14.7)	83.1 (±8)	<0.001
Gender (M/F)	144/62	46/36	0.02
BMI (kg/m^2^)	27.6 (±4)	29.4 (±15)	0.1
Number of comorbidity	1.3 (±1.1)	2.13 (±1.3)	<0.001
Charlson comorbidity index	3.1 (±2.1)	5.9 (±2.1)	<0.001

**Table 2:** Laboratory testing results in the study populations at hospital admission. Values are expressed as mean value (±SD). Hb: hemoglobin; WBC: white blood count; INR: international normalized ratio; PTT: prothrombin time; AST: aspartate amino transferase; ALT: alanine amino transferase; LDH: lactate dehydrogenase; RCP: reactive C protein; IL6: interleukin 6; BNP: brain natriuretic peptide; ALP: alkaline phosphatase; 25OHD: 25-hydroxy vitamin D.

Laboratory Tests	Alive (n = 206)	Deceased	*p*-Value
Hb (g/dL)	13.3 (±2)	12.5 (±2)	<0.001
Platelets (U/microL)	240 (±110)	200 (±90)	0.004
WBC (10^3^)	7.6 (±4)	8.7 (±5)	0.06
Neutrophils (10^3^/L)	6.1 (±4)	7.5 (±5)	0.01
Lymphocytes (10^3^/L)	1.1 (±1)	0.7(±1)	0.03
INR ratio	1.2 (±0.6)	1.3 (±0.5)	0.2
PTT (s)	30.1 (±4)	28.9 (±5)	0.06
D-dimer (ng/mL)	2769 (±5929)	6218 (±10,928)	<0.001
Fibrinogen (mg/dL) media (SD)	641 (±111)	612 (±93)	0.04
D-d/Fibrinogen media (SD)	0.6 (±0.5)	0.9 (±0.2)	<0.001
Creatinine (mg/dL) media (SD)	0.96 (±1)	1.8 (±1)	<0.001
AST (mU/mL) media (SD)	46 (±33)	123 (±539)	0.04
ALT (mU/mL) media (SD)	35.9 (±40)	55.9 (±197)	0.2
LDH (U/L) media (SD)	291 (±114)	364 (±153.4)	<0.001
PCR (mg/dL) media (SD)	8.1 (±7)	11.5 (±7)	<0.001
IL-6 (pg/mL) media (SD)	29.5 (±46)	68.1 (±76)	<0.001
Troponin (ng/mL) media (SD)	40.4 (±60)	283.9 (±762)	<0.001
BNP (pg/mL) media (SD)	280 (±1339)	532.3 (±519)	0.09
Uremia (mg/dL)	48.1 (±28)	93.5 (±54)	<0.001
ALP (U/L) media (SD)	67 (±26)	161 (±384)	<0.001
25OHD (ng/mL) media (SD)	19.6 (±14)	15.9 (±14)	0.06

**Table 3:** General characteristics and laboratory testing results in COVID-19 patients according to calcifediol supplementation. Values are expressed as mean value (±SD). BMI: Body Mass Index; RCP: reactive C protein; LDH: lactate dehydrogenase; INR: international normalized ratio; 25OHD: 25-hydroxy vitamin D; SpO2/FiO2: ratio of oxygen saturation/fraction of inspired oxygen; PaO2/FiO2: ratio of arterial oxygen partial pressure/fraction of inspired oxygen.

Characteristics	Treated with Calcifediol (n = 56)	Not Treated with Calcifediol (n = 232)	*p*-Value
Age (years)	71.8 (±16.4)	74.9 (±13.4)	0.1
BMI	29.2 (±11.5)	26.7 (±4.1)	0.008
Number of comorbidities	1.6 (±1.29)	1.6 (±1.24)	>0.9
CRP (mg/dL)	9.8 (±7.6)	8.6 (±7.2)	0.3
LDH (mU/mL)	323.2 (±148.5)	305 (±119.4)	0.3
D-dimer (ng/mL)	3812 (±8174)	3601 (±7438)	0.8
INR ratio	1.32 (±0.6)	1.23 (±1.23)	0.6
Lymphocytes (10^3^/L)	1.01 (±0.9)	1 (±0.7)	0.4
25OHD (ng/mL)	17.3 (±11.4)	18.9 (±14.3)	0.4
SpO2/FiO2	314.8 (±126.3)	333.3 (±121.3)	0.3
PaO2/FiO2	247.6 (±111.7)	239.6 (±90.9)	0.6

## Data Availability

The data presented in this study are available on request from the corresponding author. The data are not publicly available due to privacy.
